# Long-Term Engagement of Diverse Study Cohorts in Decentralized Research: Longitudinal Analysis of “All of Us” Research Program Data

**DOI:** 10.2196/56803

**Published:** 2025-03-19

**Authors:** Vijay Yadav, Elias Chaibub Neto, Megan Doerr, Abhishek Pratap, Larsson Omberg

**Affiliations:** 1 Sage Bionetworks, 2901 3rd Ave #330, Seattle, WA, 98121, United States, 1 6466838830

**Keywords:** digital health, engagement behavior, All of Us Research Program, retention, decentralized research cohorts

## Abstract

**Background:**

The generalizability of clinical research hinges on robust study designs, which include the recruitment and maintenance of a representative study population. This study examines the evolution of the demographic characteristics of 329,038 participants who enrolled and participated in The *All of Us* Research Program (AoURP), a decentralized study aimed at representing the diversity of the United States.

**Objective:**

The primary objectives of this study were to assess alterations in the demographic composition of the cohort at different protocol stages within AoURP, while analyzing completion rates and timeframes for survey and substudy completion. Additionally, we examined how participant interactions with the program impacted engagement and survey responses.

**Methods:**

We conducted a longitudinal analysis of the AoURP data, tracking changes in demographic composition, completion rates, and completion times for surveys and substudies. Comparative analyses were performed to assess differences in engagement and survey completion based on sociodemographic characteristics of participants involved in postenrollment study components.

**Results:**

The sociodemographic composition of the cohort that participated in the postenrollment study (eg, optional components) differed significantly from that of the recruited population. The proportion of self-identified White participants increased by 21.2%, whereas the proportion of Black or African American participants decreased by 12.18% (*P*=.02). Participants who identified as White (n=93,614, 52.7%) and NonHispanic (n=109,279, 42.21%) were more engaged compared to those identifying as Black or African American (n=10,887, 15.76%), Asian (n=4274, 38.72%), or Hispanic (n=12,530, 20.7%; *P*=.006). Participants’ response times to study surveys and completeness varied across all demographic groups (*P*<.001). Furthermore, those identifying as White skipped fewer survey questions (1.19) compared to those identifying as Black or African American (1.40) or other racial and ethnic identities (*P*<.001).

**Conclusions:**

The AoURP dataset serves as an exceptional resource for investigating diverse public health concerns. However, the longitudinal analysis of participant-level data underscores a significant skew in population diversity, suggesting the need for targeted strategies to enhance engagement and retention across all groups. Ensuring diversity in the cohort is essential for maintaining the study’s representativeness and the broad applicability of its findings.

## Introduction

There is a history of lack of racial, ethnic, and gender diversity in health studies [1,2]. This lack of diversity can lead to issues related to the generalizability of research findings and equity in health care. However, lack of plurality has decreased in the last three decades, partially due to regulatory and policy efforts within government agencies, such as the National Institutes of Health [3], Food and Drug Administration [4], and Department of Health Services [5], that have sought to enhance minority participation in clinical research as well as identified scientific need [6]. As the demographic makeup of the United States is becoming more pluralistic, diverse study populations are necessary to have representative and translatable observations. Much of the effort to increase minority representation in research has focused on the recruitment of diverse study populations. However, the recruited population is not always representative of the population that is ultimately studied, due to engagement rates being different across sociodemographic groups. This has been demonstrated in remote observational studies, where there is less direct interaction with participants compared to studies which require in-person visits [7]. Poor engagement can lead to study failure due to reduced sample sizes, causing loss of power or imbalanced study populations. Prior research has shown that remote-only studies demonstrate different engagement rates based on demographic features including disease presence or absence, age, race or ethnicity, and recruitment methods [6-8]. When the engagement rates vary across different populations, there is a risk that the effective study population (ie, the population with longitudinal data) becomes unrepresentative of the originally recruited cohort.

The *All of Us* Research Program (AoURP) was launched in May 2018, with an aim to recruit more than 1 million participants living in the United States to accelerate health research and precision medicine. AoURP has specifically focused on recruiting demographic categories that have historically been underrepresented in biomedical research (UBRs) and has largely succeeded in this objective through partnership with more than 340 recruitment sites nationwide [3]. During the first year of the AoURP, 80% of recruited participants self-identified as belonging to one or more UBR populations [9]. In this study, we explore long-term engagement within AoURP by exploring participation in optional components of the AoURP study (eg, surveys and substudies that could be performed post enrollment), the time that it takes participants to complete optional surveys, and survey response completeness. We hypothesize that this information, combined with data on specific interactions of participants with the AoURP can be used to improve and develop strategies that promote sustained engagement across the diverse demographics. This study primarily assesses changes in cohort demographics across AoURP protocol stages, completion rates for surveys and substudies, and the influence of participant interactions on engagement and responses. These insights provide actionable strategies for sustaining diverse, representative cohorts.

## Methods

### Study Design and Participants

**Figure 1.**
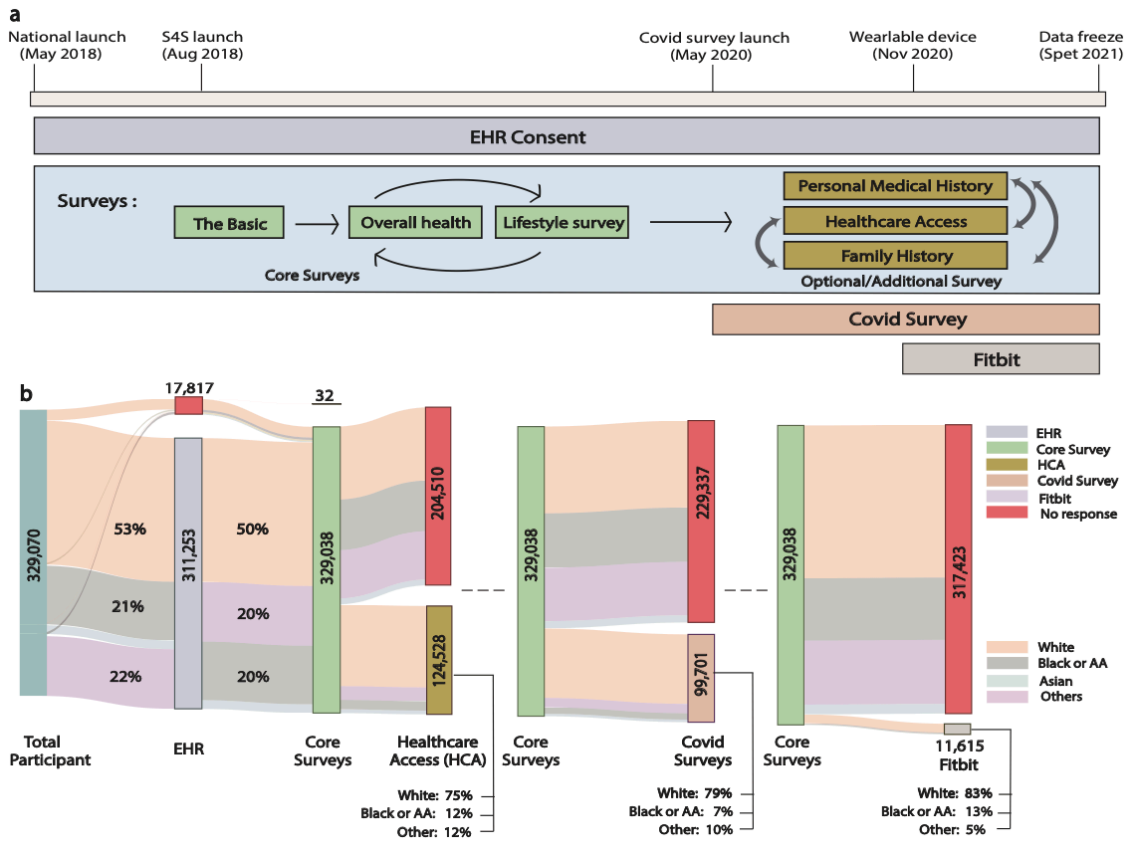
Protocol and changes in engagement across components in study. (A) Timeline for components of protocol and journey of a participant in the AoU program. Based on respective protocol launch dates, participants had the option to consent to share EHR data, participate in COVID-19 surveys, and share data from a BYOD Fitbit data. Each survey block reflects the ordering of core and additional surveys configured in the program. (B) Sankey diagram showing the population distribution within the AoURP throughout the protocol journey. The nodes represent different types of events (eg, EHR consent, completion of optional surveys, BYOD Fitbit data sharing). Flows between notes are color-coded by self-identified race (ie, White, Black, or African American, Asian, or other). The width of the nodes and links provide quantitative information. AA: African American, AoURP: *All of Us* Research Program; BYOD: bring your own device; EHR: electronic health records; HCA: Healthcare Access survey.

This study is a longitudinal study that analyzes data from participants in the AoURP, encompassing individuals aged 18 and older residing in the United States, irrespective of race, ethnicity, sex, gender, or sexual orientation [10,11]. Data from the 329,038 participants in the September 2021 AoURP data freeze were analyzed. The AoURP protocol has evolved since its launch in 2018 (Figure 1A). At present, after consenting, participants are given the option to share their electronic health record (EHR) data, provide a biosample, and answer 3 core surveys (ie, The Basics, Overall Health, and Lifestyle) [12]. Participants can also respond to additional optional health surveys including Healthcare Access (HCA), Personal Medical History (PMH), and Family Medical History (FMH), at any time and in any order after enrollment (Table S1 in Multimedia Appendix 1) [12]. In addition to these components, AoURP included two additional optional substudies, at the time of analysis: (1) a COVID-19 survey and (2) a “bring your own device” (BYOD) Fitbit study, which allows participants to share data from any Fitbit wearable device (Google Inc, Mountain View, CA) owned by them. The AoURP also collects additional data, such as physical measurements; however, our analysis is restricted to recruitment and engagement characteristics based on participant-provided information (eg, health and demographic surveys) and BYOD Fitbit data.

The core surveys included information about the participants’ self-reported demographics, which was used to explore differences in participation. Specifically, we used responses to the questions on (1) date of birth; (2) What was your biological sex assigned at birth; (3) Which categories describe you (race); (4) Which categories describe you (ethnicity); and (5) What is your annual household income (from all sources) [12]. Based on the *All of Us* (AoU) Researcher Workbench, we categorized age into three bands: younger (18‐44 years), middle aged (45‐64 years), and older adults (≥65), and household income into 4 bands (<$50,000, $50,000-$100,000, $100,000-$200,000, >$200,000). For other multiple-choice questions, we included categories with at least 1.8% respondents. This resulted in the following categories: male and female for biological sex at birth; White, Black or African American, and Asian for race; and NonHispanic and Hispanic for ethnicity. These categories were used to assess changes in cohort demographics during the course of the study, with the primary outcomes under investigation being (1) engagement (ie, number of participants who remain engaged by completing optional components after enrollment), (2) response time (ie, time between invitation and completion of optional components), and (3) completeness of response (ie, how often participants chose to answer specific questions). Understanding these changes is essential for examining shifts in participant demographics and engagement over time, which helps identify trends and biases, ensuring the cohort representativeness and development of targeted strategies for improved engagement and retention.

### Data Processing

For the primary analysis, we built an engagement cohort consisting of participants who successfully enrolled in the study and responded to the demographic questions using the AoU Researcher Workbench [13]. Since all components of the study were not launched at the same time (eg, BYOD Fitbit and the sharing of electronic health records using Sync4Science [14] were added later; see Figure 1A for the timeline), we conducted a secondary analysis using enrollment dates extracted from the observation table to evaluate the effect of timing of enrollment on these components. Specifically, this secondary analysis included individuals who enrolled after Sync4Science launch (August, 2018) and after the BYOD Fitbit protocol being launched (November, 2020). For all cohorts, activities where participants completed the surveys were considered as “active/engaged” events. All survey questions included a “prefer not to answer” option. Survey completeness was analyzed across participants by counting the number of questions answered as “prefer not to answer”.

### Statistical Analysis

Various statistical techniques were used for the three different analyses. Whenever possible we favored the use of nonparametric statistical tests and resampling techniques to avoid relying on distributional assumptions. These included the Kruskal-Wallis test (instead of analysis of variance), Mann-Whitney tests for pairwise group comparisons), bootstrapping for computation of confidence intervals, and permutation tests for calculating statistical significance. For all analyses, a significance level of *P*<.05 was used with Bonferroni correction for multiple testing [15].

To analyze engagement across self-reported age, sex at birth, race, household income, and ethnicity groups, we compared the proportion of participants responding in each demographic group between the core survey and optional components using a *χ*^2^ test for homogeneity.

Participants’ response time, as measured by the number of days between joining the study and completing (or joining) an optional component, was analyzed using the nonparametric Kruskal-Wallis one-way analysis of variance for each optional component separately, as the data was not normally distributed [16]. To further understand the variability between each group, pairwise differences between groups were analyzed using the Python language (version number 3.9; Python Software Foundation), including the *statannotation* package (version 0.4.2) [17], along with the with Mann-Whitney integrated statistical test and Bonferroni for multiple testing correction [18,19].

To better understand the relationship between various self-reported demographic variables, a linear mixed model (LMM) was used to analyze the response time across the 3 surveys concomitantly. To improve the fit of the model, response time data was log-transformed before feeding into the model. All self-reported demographic variables were fitted into the model to evaluate the effect produced by each predictor. Age was treated as a continuous variable, while other variables were fitted as categorical variables. The coefficient of relative change for each variable was reported by the LMM model, and the log-transformed relative change between each group was back-transformed to a percentage change in response time across groups for all the categories, using formula 100(e^coefficient^–1). To further evaluate confidence intervals, bootstrapping was used. To account for the repeated measurements of each subject, the bootstrap confidence intervals for the LMMs were constructed based on 1000 iterations of the following couple of steps. First, we obtained a bootstrap sample from the original data by sampling with replacement at the subject level (ie, assuming that our dataset contains data from *N* subjects, we grouped the repeated measurements of each subject as a “subject block,” and then sampled with replacement N “subject blocks” out of the N blocks in the original dataset.) Second, for each bootstrap sample, we fit a LMM and computed the coefficient of relative change of each variable as described above. Finally, we computed 95% CI for the coefficients of relative change using the percentile interval method, where the lower and upper confidence bounds correspond to the 0.025 and 0.975 quantiles of the distribution of coefficients generated across the 1000 bootstrap iterations.

We independently examined outliers in the response time for optional surveys to check if these outliers were the cause of any systematic differences. Outliers comprised any individual who had a response time less than the first quartile (Q1)–1.5×IQR or greater than Q3+1.5×IQR. To evaluate the differences, we computed the proportion of outlier participants for each demographic group and then conducted a *χ*^2^ test (test of homogeneity) for testing the equality of outlier participants proportion across different demographic groups.

Completeness of response was evaluated by counting the number of questions where a participant responded “prefer not to answer.” First, the nonparametric Kruskal-Wallis test was used to evaluate variability across different groups (since the data was not normally distributed). However, due to ties in the completeness of the response variable, we adopted a permutation test to assess differences between the groups [12]. The permutation test was conducted by comparing the observed value of the ANOVA F-statistic computed in the original data (F_obs_) against the permutation null distribution of the F-statistic computed on permuted versions of the data (F*), where the group labels were randomly shuffled. The test was based on 1000 data permutations and the permutation *P* value was computed as the proportion of times the permuted F-statistic was greater or equal to the observed test statistic (ie, *P*=(1+ sum{i=1..B indicator{F*>=F_obs_})/(1+ B), for B=1000). Due to the large sample set, it is possible that we may be detecting very small effect sizes with very high statistical significance across different categories. To assess the effect size, 95% CI were computed using nonparametric bootstrapping for the pairwise group mean difference for each group [20].

### Ethical Considerations

This study used deidentified data from the *All of Us* Research Program, which obtained institutional review board (IRB) approval and informed consent from participants for secondary research [21]. The original data collection was approved by the AoURP IRB [21], and no additional IRB approval was required for this secondary analysis. According to the All of Us Responsible Conduct of Research training, analyses using deidentified data on the Researcher Workbench do not require a separate IRB review [22], as the research involves no direct interaction with participants. All analyses adhered to ethical principles, ensuring privacy and confidentiality as outlined by the AoURP.

## Results

### Population Characteristics

Data from 329,078 consented participants were made available as part of the version 5 data release [23] by AoURP. Of the total consented participants, 329,038 (99.98%) completed all core surveys and were considered successfully enrolled in the program. Additionally, 311,253 participants (94.59%) consented to share all their electronic health records; including retrospective medical records from before the AoURP study was launched. Post completion of core surveys, participants had the option to complete three optional health surveys. A significantly smaller proportion completed these three optional surveys: (1) HCA-37.84% (n=124,528/329,078), (2) FMH-117,693 (35.76%), and (3) PMH-113,830 (34.59%) (Figure 1B). Both the COVID survey and the BYOD Fitbit data collection were launched at later stages of the study (Figure 1A). A total of 99,701 (30.3%) people participated in the first COVID survey and 11,615 (3.52%) participated in BYOD Fitbit data sharing. Participants who enrolled before the BYOD Fitbit data collection protocol was launched were less likely to consent to share their BYOD Fitbit data compared to those who enrolled after the protocol launch (3.51% vs 6.51%, respectively).

**Figure 2.**
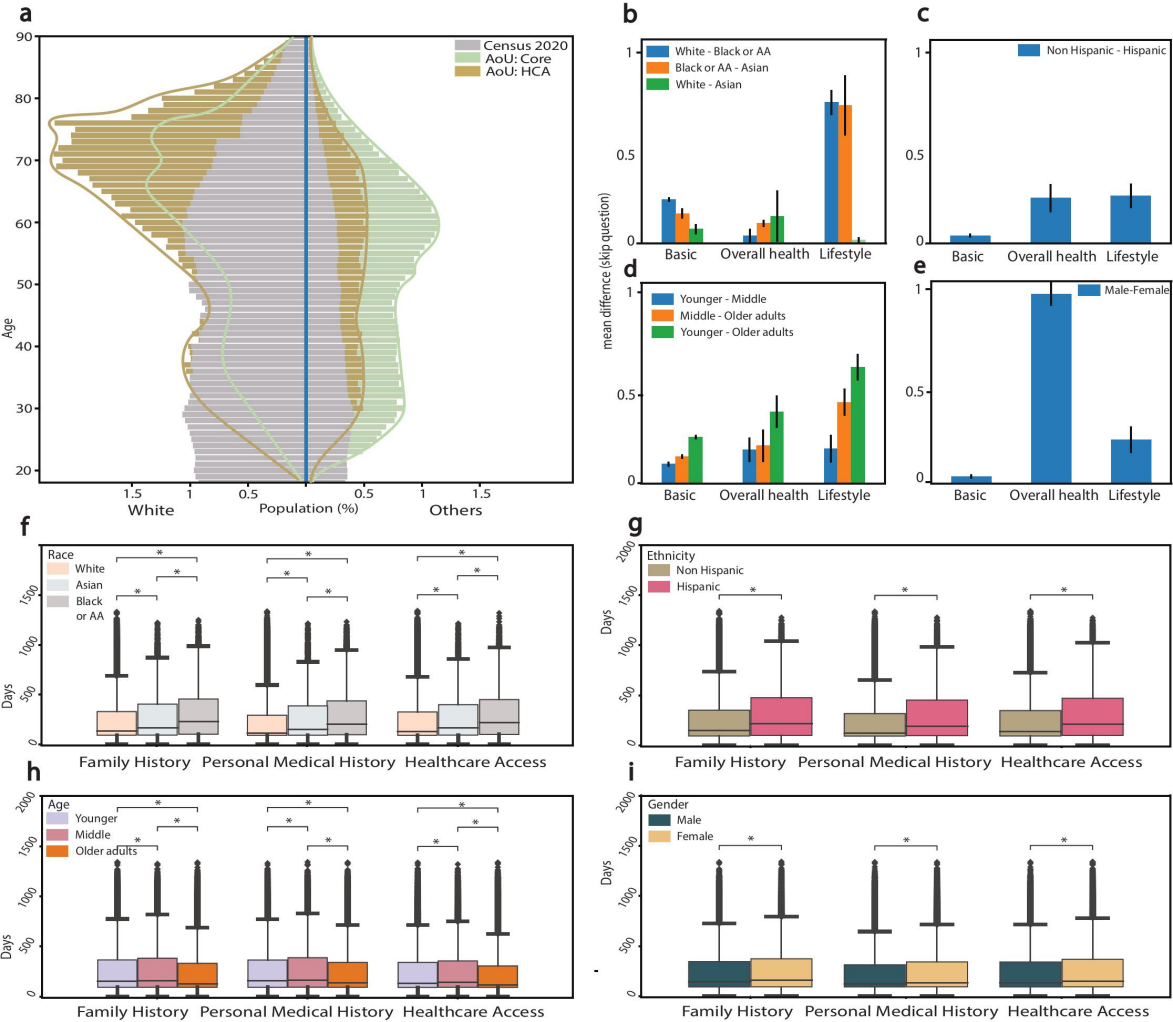
(a) Population pyramids divided by self-reported race (White vs other) see Multimedia Appendix 1 for additional populations pyramids. Three different pyramids are displayed for comparison for three different populations representing: those who responded to: AoURP core survey, one optional survey (HCA) and the 2020 US census. At baseline, based on response to the core survey) cohorts tend to be older and less white compared to the 2020 US Census, while responses to later optional surveys showed that the proportion of self-reported White participants increased significantly and a sudden drop in participants self-identifying from other racial groups. (B-E) represents the mean difference of number of skipped questions between demographic groups specific to each survey for: (b) *Race,* (c)*: Ethnicity,* (d)*: Age,* (e)*: Sex at birth*. The bar plot with the error bar represents the bootstrap (1000 iterations) 95% confidence interval for pairwise group mean differences. (f): Race, (g): Ethnicity, (h): Age and (i): Sex at birth) represent pairwise statistically significant differences (*P*<.001) across all the self-reported demographic groups of the participants responded to the additional surveys. AA: African American; AoU: All of Us. HCA: Health Care Access. *P<.001.

The AoURP aims to recruit persons from the UBR populations, and recruitment was successful, with 24.36% (n=80,154) of the participants responding to the core surveys with a self-reported race of Black or African American and Asian, while 53.98% (n=177,615) self-reported as White. We compared the distribution of ages and race to the 2020 US Census data [24], which showed the enrichment of self-identified races other than White, as well as middle-aged individuals, in the AoU cohort (Figure 2A) at baseline. Additionally, more participants reported being female (Figure S1a in Multimedia Appendix 1) and Hispanic ethnicity (Figure S1b in Multimedia Appendix 1) compared to the census.(However, this initial enrichment of people self-identifying as belonging to one or more UBR populations diminished as participants engaged with optional components of the protocol. Distributions across demographic variables for each component of the study is shown in Table 1. Moreover, a total of 1416 (0.4%) deaths were reported by the end of version 5 data release.

**Table 1.** Demographics characteristics and percentage distribution of participants in each component.

Groups	Core survey^a^ (n=329,038), n (%)	HCA^b^ survey (n=124,528), n (%)	Family history (n=117,693), n (%)	Medical history (n=113,830), n (%)	EHR^c^ (n=271,421), n (%)	Covid survey (n=99,701), n (%)	Fitbit (n=11,615), n (%)
Age (years)							
18‐44	108,254 (32.9)	35,740 (28.7)	33,307 (28.3)	32,328 (28.4)	88,212 (32.5)	23,130 (23.2)	3,984 (34.3)
45‐64	121,415 (36.9)	42,090 (33.8)	39,545 (33.6)	38,019 (33.4)	100,697 (37.1)	33,698 (33.8)	4,321 (37.2)
≥65	99,369 (30.2)	46,698 (37.5)	44,841 (38.1)	43,483 (38.2)	82,512 (30.4)	42,971 (43.0)	3310 (28.6)
Sex							
Male	125,034 (38)	42,090 (33.8)	39,663 (33.7)	38,475 (33.8)	104,497 (38.5)	33,699 (33.8)	3,357 (28.9)
Female	199,726 (60.7)	81,441 (65.4)	77,207 (65.6)	74,559 (65.5)	163,395 (60.2)	65,304 (65.5)	8,200 (70.6)
Race							
White	177,681 (54)	93,645 (75.2)	89,094 (75.7)	86,625 (76.1)	144,124 (53.1)	78,963 (79.2)	9,698 (83.5)
African American	69,098 (21)	10,958 (8.8)	10,004 (8.5)	9,448 (8.3)	58,898 (21.7)	7,278 (7.3)	581 (5)
Asian	11,187 (3.4)	4,234 (3.4)	4,002 (3.4)	3,870 (3.4)	8,685 (3.2)	2,892 (2.9)	372 (3.2)
Ethnicity							
NonHispanic	258,953 (78.7)	109,336 (87.8)	103,570 (88)	100,398 (88.2)	212,523 (78.3)	89,631 (89.9)	10,674 (91.9)
Hispanic	60,543 (18.4)	12,577 (10.1)	11,652 (9.9)	11,041 (9.7)	51,027 (18.8)	7,876 (7.9)	767 (6.6)
Income (USD)							
<50,000	139,183 (42.3)	38,106 (30.6)	35,661 (30.3)	34,035 (29.9)	116,982 (43.1)	27,916 (28)	2,532 (21.8)
50,000-100,000	59,556 (18.1)	32,626 (26.2)	31,071 (26.4)	30,279 (26.6)	48,041 (17.7)	27,518 (27.6)	3,554 (30.6)
100,000-200,000	46,394 (14.1)	28,766 (23.1)	27,422 (23.3)	26,750 (23.5)	36,913 (13.6)	24,526 (24.6)	3,520 (30.3)
>200,000	20,071 (6.1)	12,702 (10.2)	12,005 (10.2)	11,724 (10.3)	15,851 (5.84)	10,469 (10.5)	1,371 (11.8)

a Core Survey at enrollment.

b HCA: Health Care Access.

c EHR: electronic health records.

### Differences in Engagement

The relative shift in the proportions of participants is reported as percentage changes. We observed an increase of 21.2% in the proportion of self-identified White participants and correspondingly a decrease of Black or African American respondents by 12.18% in the HCA compared to the Core surveys. Similarly, the proportion of participants who identified as NonHispanic (of all races) increased by 9.1%, to 87.8% (n=109,336) of the total respondent pool in the HCA survey, while people self-identifying as Hispanic decreased by 8.33% to 10.1% of the respondent pool (Table 1 and Figure 2A). To explore the engagement further, we compared the percentage of each self-identified demographic group that continued in each optional component of the study (ie, optional surveys, EHR consent, COVID Surveys, and BYOD Fitbit) and observed that the engagement varied across most components except the BYOD Fitbit (Table 2). The engagement with optional surveys was 3 times higher among people who identified as White, with 52.7% (n=93,614) of White participants who enrolled and also completed the optional surveys (HCA), compared to Black or African American participants (n=10,887, 15.8%), and nearly twice as high as those identifying as Asian (n=12531, 38.7%) (*P*<.001). Similarly, 42.2% (n=109,279) of those self-identifying as nonHispanic (of all races) engaged with optional surveys as compared to 20.7% (n=12,531) of self-identified Hispanic participants (*P*<.006). 63.4% (n=12,681) of participants who self-identified with high household income engaged in the optional surveys compared to 27.4% (n=38,190) of self-identified with low household income (*P*<.001). A total of 47% (n=46,743) of older adults (aged >65 years) engaged with optional surveys compared to 34.64% (n=37,504) and 33.05% (n=40,096)of the middle-aged and younger groups, respectively. Engagement was 40.8% (n=81,460) for participants who self-identified as female at birth engaged in the optional surveys compared to 33.66% (n=42,137) of self-identified male at birth. However, neither self-reported age nor sex at birth were statistically significant indicators of engagement using the *χ*^2^ test.

The first COVID survey showed large differences in participation across age (*P*=.02), race (*P*<.001), household income (*P*<.001), and ethnicity (*P*<.001), but not by sex at birth (*P*=.45), Older individuals participated at higher rates, with 43.2% (n=42,964) of self-reported older adults compared to 27.8% (30,099) of middle-aged and 21.3% (n=25,841) of younger participants completing the first survey. Self-reported White participants (n=79,048, 44.5%) participated at more than 4 times higher levels than self-identified Black or African American participants (n=7281, 10.54%) and nearly twice those identifying as Asian (n=2880, 26.09%). Self-identified high-income participants (n=10,485, 52.4%) participated at more than approximately 2.5 times higher levels than self-identified low household income participants (n=27,876, 20%). Similarly, participants self-identifying as nonHispanic (n=89,552, 34.59%) engaged at almost 3 times higher rates than those identifying as Hispanic (n=7876, 13.01%).

We did not observe any statistically significant differences in engagement by self-reported age, race, ethnicity, or sex in participation rates for the BYOD Fitbit substudy or the consent to share EHR data.

**Table 2.** Demographics characteristics and response rate proportion specific to each survey in comparison to core surveys, (ie, response rate percentage = Number of participant responded to optional surveys/Number of participants responded to core survey)×100.

Groups	Core^a^ (n)	HCA^b^ survey, (%)	*P* value	Family history (%)	*P* value	Medical history, n (%)	*P* value	EHR^c^, n (%)	*P* value	Covid survey, n (%)	*P* value	Fitbit^d^, n (%)	*P* value
Age groups (years)			.22		.18		.19		.94		.02		.99
18‐44	121,317	40,156 (33.1)		37,244 (30.7)		36,274 (29.9)		96,932 (79.9)		25,841 (21.3)		4,489 (3.7)	
45‐64	108,268	37,461 (34.6)		35,295 (32.6)		33,996 (31.4)		91,162 (84.2)		30,099 (27.8)		3,898 (3.6)	
>65	99,453	46,743 (47)		44,853 (45.1)		43,461 (43.7)		82,745 (83.2)		42,964 (43.2)		3,282 (3.3)	
Sex			.41		.41		.42		.81		.45		.59
Male	125,184	42,187 (33.7)		39,683 (31.7)		38,431 (30.7)		105,530 (84.3)		33,674 (26.9)		3,380 (2.7)	
Female	199,658	81,460 (40.8)		77,068 (38.6)		74,672 (37.4)		162,122 (81.2)		65,288 (32.7)		8,186 (4.1)	
Race			<.001		<.001		<.001		0.40		<.001		.18
White	177,637	93,615 (52.7)		89,174 (50.2)		86,509 (48.7)		135,359 (76.2)		79,048 (44.5)		9,770 (5.5)	
African American	69,084	10,915 (15.8)		10,017 (14.5)		9,395 (13.6)		63,557 (92)		7,254 (10.5)		553 (0.8)	
Asian	11,040	4,272 (38.7)		3,974 (36)		3,875 (35.1)		8,578 (77.7)		2,881 (26.1)		364 (3.3)	
Ethnicity			.006		.006		.006		.41		.001		.23
NonHispanic	258,895	109,254 (42.2)		103,558 (40)		100,451 (38.8)		208,152 (80.4)		89,578 (34.6)		10,615 (4.1)	
Hispanic	60,535	12,531 (20.7)		11,623 (19.2)		11,078 (18.3)		55,147 (91.1)		7,870 (13)		787 (1.3)	
Income (USD)			.001		.001		.001		.98		<.001		.31
<50,000	139,380	38,162 (27.4)		35,639 (25.6)		34,092 (24.5)		134,125 (96.2)		27,946 (20.1)		2,537 (1.8)	
50,000- 100,000	59,563	32,700 (54.9)		31,134 (52.3)		30,222 (50.7)		55,078 (92.5)		27,554 (46.3)		3,556 (6)	
100,000- 200,000	46,381	28,728 (61.9)		27,411 (59.1)		26,729 (57.6)		42,346 (91.3)		24,512 (52.9)		3,520 (7.6)	
>200,000	20,008	12,685 (63.4)		12,043 (60.2)		11,785 (58.9)		18,181 (90.9)		10,494 (52.5)		1,377 (6.9)	

a Core survey at enrollment.

b HCA: Health Care Access.

c EHR: electronic health records.

d Fitbit was launched later in the study (see Figure 1). For a comparison of engagement rates between participants who joined before and after the launch of the Fitbit component (see Table S5 in Multimedia Appendix 1).

### Response Time to Optional Surveys

In addition to differences in engagement, we also analyzed how long it took for participants to participate in optional surveys as measured by the time in days between enrollment and completion of the optional component. Given that only the three optional surveys (ie, HCA, PMH, and FMH) were available, and the COVID Surveys and BYOD Fitbit, were unavailable at the launch of the study, we were only able to explore the delay for these surveys. We found differences in the response time based on self-reported age, sex at birth, race, ethnicity, and household income (all *Ps*<.001 based on Kruskal-Wallis one way analysis of variance). These results are summarized in Table S2 in Multimedia Appendix 1. Overall for the optional surveys, response times ranged from 0 to 1342 days with a mean of 247.9 (SD 233) days and median value was 141 (IQR 314.55) days. Self-reported older adults (median 117, IQR 213) took less time to respond to optional surveys compared to younger (median 135, IQR 251) and middle aged (median 138, IQR259) participants. People who identified as male at birth (median 120, IQR 221) took less time compared to people who self-identified as female at birth (median 133, IQR249). People who self-identified as White completed surveys sooner after enrollment (median 115, IQR 202) in comparison to those who self-identified as Black or African American (median 202, IQR 340), and Asian (median 153, IQR 295). People who identified as nonHispanic of any race (median 123, IQR 225) responded sooner to optional surveys as compared to people who self-identified as Hispanic (of all races) (median 188, IQR 335). People self-identified with high household income (median 119, IQR 195) took less time compared to people with low household income (median 150, IQR 279). We performed a secondary analysis to determine pairwise differences using the nonparametric Mann-Whitney test to illustrate which groups had quicker participation (Figure 2F–I). The linear mixed effect results presented in Table 3 provided valuable insights into the relationships between various demographic variables and their impact on response time. The table displays the estimated effects in terms of coefficients and relative percentage changes, with the use of bootstrap for accuracy. When examining age groups, individuals aged 45-64 years exhibited a 3.04% increase in response time compared to the reference group (18-44 years), while those aged ≥65 years displayed a decrease of −2.76%. Gender differences were also notable, with women showing a 5.65% increase in response time compared to men. In terms of race, African Americans exhibited a substantial 33.5% increase in response time compared to White individuals, while Asians and other racial groups also displayed significant differences. Ethnicity played a role as well, with Hispanics experiencing an 18.41% increase ( Table 3) . Finally, income levels showed a consistent trend, with higher income brackets associated with decreased response times, highlighting the influence of socioeconomic factors on response behavior. These results offer important insights into the complex interplay between demographics and response times, shedding light on potential areas for further investigation and intervention. We conducted a secondary analysis to check if outliers were responsible for any significant difference and found no difference in the demographics. Distribution across demographic data is shown in Table S3 in Multimedia Appendix 1. We also explored the interaction effect (see Table S4 in Multimedia Appendix 1) between other demographic features and household income. The study found that the effect of age, gender, race, and ethnicity on response time was moderated by income. The response time was slower for people with higher income in all age groups, except 18-44 years. The difference was most pronounced for people aged ≥65 years. The interaction effect between gender and income was only significant for people who identify as neither male nor female. The interaction effect between race and income was significant for all race groups except White. The interaction effect between ethnicity and income was only significant for people who were not in plurality.

**Table 3.** Estimated effect (coefficient and relative % change using bootstrap) on response time.

Variables	Model	
	Coefficient	Percent change (95% CI)
Age (years)		
18-44	0	0
45-64	0.03	3.04 (2.99-3.09)
>65	–0.028	–2.76 (–2.78 to –2.69)
Gender		
Male	0	0
Female	0.055	5.65 (5.55-5.65)
Race		
White	0	0
African American	0.289	33.50 (33.40-33.57)
Asian	0.169	18.41 (18.30-18.54)
Ethnicity		
NonHispanic	0	0
Hispanic	0.169	18.41 (18.26-18.49)
Income (USD)		
<50,000	0	0
50,000-100,000	–0.045	–4.79 (–4.91 to –4.56)
100,000-200,000	–0.066	–6.39 (–6.45 to –6.31)
>200,000	–0.05	–4.88 (–4.96 to –4.78)

### Completeness of Responses

To evaluate the frequency of skipped questions in each survey (survey completeness), we counted skipped questions and compared those across self-reported demographic groups (Table S5 in Multimedia Appendix 1). Skipped questions were relatively rare, with approximately 124,000 skipped questions out of 34 million answered; on average 329,038 participants skipped a mean of 1.32 (SD 0.74) questions per survey. We reported the results of “completeness of response” based on core surveys only, given that all the questions in the optional surveys were already answered except for one participant, who skipped one question. Using a nonparametric test, we observed differences in completeness for all self-reported demographic groups in the three core surveys. Mean values are reported for missing data (ie, skipped questions). Middle-aged participants skipped more questions ( 1.72 questions) compared to 1.54 for younger and 1.6 for seniors (*P*<.001). Participants who identified as female at birth skipped fewer questions (1.58) compared to participants who identified male at birth (1.99) (*P*<.001). Participants who self-reported as Black or African American race (1.86) and people self-reporting nonHispanic ethnicity of any race (1.71) skipped more questions compared to people of self-identified White (1.62) and Asian (1.62) race, and those who self-reported Hispanic (1.57) ethnicity of any race (*P*<.001). Participants with high household income skipped fewer questions (1.58) compared to participants with low household income (1.61) (*P*<.001). Also, we conducted pairwise group mean differences to further estimate the effect size which returned significant results (Figure 2B-E).

### Secondary Analysis on Effect of Timing of Enrollment on Engagement

As all protocols were not launched at the same time, time of enrollment may have affected the engagement behavior of the participants who joined before and after the launch of the specific study components. We conducted a secondary analysis to evaluate if there were any differences in engagement between individuals who enrolled before and after the launch of BYOD Fitbit and Sync4Science separately. We found no significant differences in the demographic groups. Distribution across demographic variables for pre- and postlaunch of BYOD Fitbit (Table S6 in Multimedia Appendix 1), and for Sync4Science is shown in Table S7 in Multimedia Appendix 1.

## Discussion

### Principal Finding

The utility and translatability of studies is dependent on the study being carried out on a representative population. The AoURP has enrolled an extremely large cohort that aims to be representative of the diversity of the broader US population [25]. However, the data collected over time is not as representative as the enrolled population, with differences in engagement across demographic groups. This pattern is observed in both, the demographics that remained engaged and the degree to which participants completed optional substudy components and how quickly they responded [26]. These variations are driven by socioeconomic, cultural, and structural factors, underscoring the need for tailored strategies to mitigate participation disparities [27]. Addressing these disparities is essential to maintain the cohort’s representativeness, which directly impacts the validity and generalizability of the findings [28].

### Comparison to Prior Work

This study builds on prior research by highlighting demographic engagement disparities in longitudinal cohort studies, an issue that has been well-documented in other population-based research efforts [29,30]. Previous studies have reported similar challenges, such as the underrepresentation of minority groups and the overrepresentation of higher socioeconomic status in voluntary participation metrics [31]. However, unlike earlier research, this study leverages the unique scale and diversity of the AoURP cohort to provide deeper insights into how engagement patterns vary over time and across demographic groups. By emphasizing socioeconomic and structural drivers of engagement disparities, this work highlights the need for target interventions necessary for retaining participants and ensuring equitable representation [32].

### Strengths and Limitations

Our study benefits from a large, diverse cohort and longitudinal data, providing valuable insights into participation patterns. However, there are limitations that must be acknowledged. First, there may be biases due to self-selection and varying dropout rates among demographic groups. Overrepresentation of more engaged participants with higher socioeconomic status in optional surveys could skew health outcome estimates [7]. Second, the high dropout rates observed among underrepresented groups over time pose a challenge to maintaining sample diversity, which could limit the generalizability of findings to the broader US population [33]. Third, reliance on self-reported data, which may introduce reporting biases that affect the accuracy of the findings [27]. To address these limitations, efforts should focus on implementing strategies such as over-recruiting populations with higher attrition rates, developing tailored retention programs, and validating self-reported data through objective measures such as linked medical records [29]. These steps can enhance the reliability and applicability of the study’s findings, ensuring their broader impact.

### Future Directions

Based on our findings, decentralized studies such as AoURP should prioritize interventions that address engagement disparities. Over-recruiting populations with higher attrition rates and implementing targeted outreach strategies for underrepresented groups can enhance participation. Importantly, these strategies should be codesigned with input from participants to address specific barriers effectively [30]. Addressing challenges such as the digital divide, cultural differences, and logistical barriers is critical to fostering equity in research participation [31,34]. Innovative approaches such as bidirectional engagement where participants actively contribute to study design and receive meaningful feedback could further strengthen retention. Additionally, leveraging digital health tools and adaptive technologies can facilitate participant engagement while addressing socioeconomic barriers [35].

### Conclusion

This study underscores the importance of maintaining representativeness in large, decentralized cohort studies such as AoURP. By identifying participation disparities and their underlying drivers, we provide actionable insights to improve cohort retention and engagement strategies. Future research should continue to explore and validate these interventions to ensure equitable and impactful scientific discoveries that benefit all segments of the population.

## Supplementary material

10.2196/56803 Multimedia Appendix 1 Demographic characteristics.

## Abbreviations

AoU: All of Us
AoURP: All of Us Research Program
BYOD: bring your own device
EHR: electronic health record
FMH: Family Medical History
HCA: Health Care Access
IRB: institutional review board
LMM: linear mixed model
PMH: Personal Medical History
UBR: underrepresented in biomedical research
