# Supplementary material for: Long-Term Engagement of Diverse Study Cohorts in Decentralized Research: Longitudinal Analysis of “All of Us” Research Program Data
Source: Interact J Med Res. 2025 Mar 19;14:e56803. doi: 10.2196/56803 (PMC11966662; doi:10.2196/56803)
Supplement: Multimedia Appendix 1 [file ijmr-v14-e56803-s001.docx]

Table S1: Survey question summary

Figure S1 A-B: Population pyramids

Table S2: Demographics characteristics and response time (number of days) specific to each survey

Table S3: Demographics characteristics and outlier percentage distribution of participants specific to optional survey

Table S4: Estimated interaction effect (coefficient and relative % change using bootstrap) on response time

Table S5: Demographics characteristics and distribution of median skip questions count using permutation test. (Reporting results for core surveys only as for optional none of the questions were skipped

Table S6: Demographics characteristics and response rate proportion of population who joined before and after the first wearable device (Fitbit) protocol was deployed (Nov 2020)

Table S7: Demographics characteristics and response rate proportion of population who joined before and after EHR integration ('Sync4Science' protocol) was deployed.

| **Table S1: Survey question summary** | | |
| --- | --- | --- |
|  | | |
| **Survey** | **Number of Questions** | **Survey Type** |
| The Basic | 23 | Core |
| Overall Health | 24 | Core |
| Lifestyle | 31 | Core |
| Health Care Access | 462 | Additional or Optional |
| Personal Medical History | 57 | Additional or Optional |
| Family Medical History | 67 | Additional or Optional |

**Figure S1. (A). Population pyramids divided by self-reported gender (Male vs Female); (B) Population pyramids divided by self-reported Ethnicity (Non- vs Hispanic). Three different pyramids are displayed for comparison for three different populations representing: those who responded to: AoURP core survey, one optional survey (HCA) and the 2020 US census.**
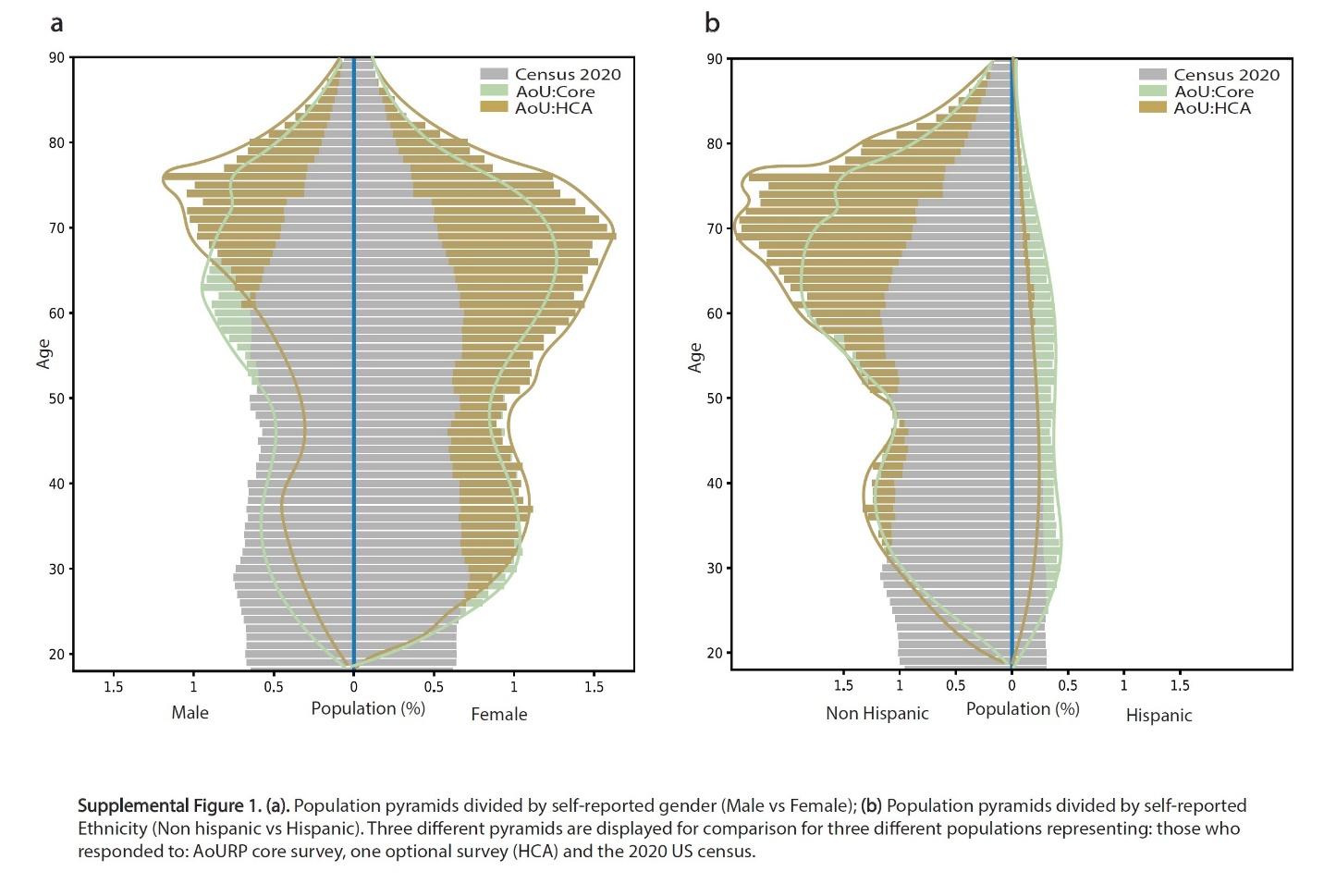


|  |  |  |  |  |  |  |  |  |
| --- | --- | --- | --- | --- | --- | --- | --- | --- |
| **Table S2: Demographic characteristics and response time(number of days) specific to each survey** | | | | | | |  |  |
|  | Groups | HCA (days)  Median(IQR) | P value | Medical History (days)  Median(IQR) | P value | Family History (days)  Median(IQR) | P value |  |
| Age Group | 18-44 | 135 (251) | 3.2e- 43 | 157 (274) | 4.8e- 47 | 152 (274) | 1.2e- 56 |  |
|  | 45-64 | 138 (259) |  | 167 (291) |  | 156 (287) |  |  |
|  | 65> | 117 (213) |  | 136 (247) |  | 127 (239) |  |  |
| Sex | Male | 120 (221) | 7.1e- 34 | 142 (253) | 6.7e- 34 | 134 (249) | 5.5e- 32 |  |
|  | Female | 133 (249) |  | 157 (280) |  | 150 (275) |  |  |
| Race | White | 115 (202) | 0 | 136 (238) | 0 | 128 (235) | 0 |  |
|  | African American | 202 (340) |  | 228 (354) |  | 219 (350) |  |  |
|  | Asian | 153 (295) |  | 168 (311) |  | 164 (306) |  |  |
| Ethnicity | Non- Hisp | 123 (225) | 5.2e- 206 | 147 (258) | 4.2e- 182 | 137 (254) | 3.6e- 194 |  |
|  | Hisp | 188 (355) |  | 216 (377) |  | 211 (371) |  |  |
| Income | <50k | 168 (308) | 1.8e- 113 | 150 (279) | 4.4e- 125 | 165 (304) | 2.9e- 122 |  |
|  | 50k-100k | 140 (250) |  | 117 (217) |  | 131 (247) |  |  |
|  | 100k-200k | 138 (234) |  | 117 (198) |  | 129 (229) |  |  |
|  | 200k> | 139 (235) |  | 119 (195) |  | 131 (228) |  |  |

|  |  |  |  |  |  |  |  |
| --- | --- | --- | --- | --- | --- | --- | --- |
| **Table S3: Demographic characteristics and outlier percentage distribution of participants specific to optional survey** | | | | | | |  |
|  | Groups (n) | Medical History  n% | P value | HCA  n% | P value | Family History  n% | P value |
| Age Group | 18-44 | 5.06 | 0.77 | 6.18 | 0.73 | 5.11 | 0.74 |
|  | 45-64 | 3.99 |  | 5.59 |  | 4.22 |  |
|  | 65> | 6.27 |  | 8.29 |  | 6.69 |  |
| Sex | Male | 6.05 | 0.79 | 7.78 | 0.58 | 6.39 | 0.62 |
|  | Female | 4.48 |  | 6.2 |  | 4.76 |  |
| Race | White | 6.97 | 0.11 | 9 | 0.06 | 7.14 | 0.12 |
|  | African  American | 1.2 |  | 1.54 |  | 1.39 |  |
|  | Asian | 3.48 |  | 4.16 |  | 3.89 |  |
| Ethnicity | Non- Hisp | 5.66 | 0.16 | 7.5 | 0.09 | 6.01 | 0.15 |
|  | Hisp | 1.11 |  | 1.58 |  | 1.2 |  |
| Income | <50k | 3.16 | 0.43 | 4.27 | 0.27 | 3.17 | 0.35 |
|  | 50k to 100k | 7.61 |  | 10.21 |  | 8.14 |  |
|  | 100k to 200k | 2.89 |  | 3.6 |  | 2.85 |  |
|  | 200k> | 7.29 |  | 9.45 |  | 7.69 |  |
| Note: n% : percentage distribution of subjects in each survey | | | |  |  |  |  |
|  |  |  |  |  |  |  |  |

**Table S4: Estimated interaction effect (coefficient & relative % change using bootstrap) on response time.**

|  | **Model** | |
| --- | --- | --- |
| Variables | Coefficient | % change (95% CI) |
| **Age X Income** |  |  |
| 18 to 44 X Less than 50k | 0 | 0 |
| 45 to 64 X From 50k to 100k | -0.01 | -1 (-1.17 to -0.92) |
| 65 above X From 50k to 100k | -0.027 | -2.67 (-2.98 to -2.35) |
| 45 to 64 X From 100k to 200k | 0.046 | 4.7 (4.27 to 5.11) |
| 65 above X From 100k to 200k | 0.042 | 4.28 (3.97 to 4.39) |
| 45 to 64 X More than 200k | 0.052 | 5.33 (5.17 to 5.41) |
| 65 above X More than 200k | 0.114 | 12.07 (11.86 to 12.43) |
| **Gender X Income** |  |  |
| Male X Less than 50k | 0 | 0 |
| Female X From 50k to 100k | 0.012 | 1.2 (-1.11 to 1.37) |
| Others X From 50k to 100k | -0.067 | -6.49 (-6.56 to -2.40) |
| Female X From 100k to 200k | -0.01 | -1 (-1.09 to -0.88) |
| Others X From 100k to 200k | -0.13 | -12.2 (-12.41 to -12.15) |
| Female X More than 200k | -0.075 | -7.33 (-7.37 to -7.29) |
| Others X More than 200k | -0.243 | -11.58 (-11.66 to -11.49) |
| **Race X Income** |  |  |
| White X Less than 50k | 0 | 0 |
| African American X From 50k to 100k | -0.061 | -5.92 (-6.01 to -5.88) |
| Asian X From 50k to 100k | 0.009 | 0.9 (0.9 to 1.15) |
| Others X From 50k to 100k | -0.062 | -6.02 (-6.14 to -5.97) |
| African American X From 100k to 200k | -0.058 | -5.64 (-5.68 to -5.54) |
| Asian X From 100k to 200k | 0.084 | 8.76 (8.61 to 8.77) |
| Others X From 100k to 200k | -0.107 | -10.15 (-10.27 to -10.12) |
| African American X More than 200k | -0.119 | -11.22 (-11.25 to -11.13) |
| Asian X More than 200k | -0.024 | -2.38 (-2.45 to -2.37) |
| Others X More than 200k | -0.042 | -4.12 (-4.15 to -4.05) |
| **Ethnicity X Income** |  |  |
| NonHispanic X Less than 50k | 0 | 0 |
| Hispanic X From 50k to 100k | 0.012 | 1.2 (1.18 to 1.27) |
| Others X From 50k to 100k | -0.05 | -4.88 (-4.91 to -4.81) |
| Hispanic X From 100k to 200k | 0.015 | 1.51 (1.44 to 1.52) |
| Others X From 100k to 200k | -0.103 | -9.79 (-9.84 to -9.78) |
| Hispanic X More than 200k | -0.075 | -7.23 (-7.29 to -7.19) |
| Others X More than 200k | 0.06 | 6.18 (6.09 to 6.24) |

|  |  |  |  |  |  |  |  |
| --- | --- | --- | --- | --- | --- | --- | --- |
| **Table S5: Demographic characteristics and distribution of median skip questions count using permutation test. (Reporting results for core surveys only as for optional none of the questions were skipped** | | | | | | | |
|  | Groups | Basic  Mean | P value | Overall Health  Mean | P value | Lifestyle  Mean | P value |
| Age Group | 18-44 | 1.41 | <0.001 | 1.66 | <0.001 | 1.56 | <0.001 |
|  | 45-64 | 1.32 |  | 1.83 |  | 2.01 |  |
|  | 65> | 1.18 |  | 2.02 |  | 1.6 |  |
| Sex | Male | 1.31 | <0.001 | 2.59 | <0.001 | 2.09 | <0.001 |
|  | Female | 1.27 |  | 1.62 |  | 1.87 |  |
| Race | White | 1.19 | <0.001 | 1.82 | <0.001 | 1.65 | <0.001 |
|  | African  American | 1.4 |  | 1.86 |  | 2.34 |  |
|  | Asian | 1.25 |  | 1.96 |  | 1.67 |  |
| Ethnicity | Non- Hisp | 1.27 | <0.001 | 1.85 | <0.001 | 2.01 | <0.001 |
|  | Hisp | 1.31 |  | 1.63 |  | 1.77 |  |
| Income | <50k | 1.24 | <0.001 | 1.69 | <0.001 | 1.90 | <0.001 |
|  | 50k to 100k | 1.11 |  | 1.83 |  | 1.41 |  |
|  | 100k to 200k | 1.08 |  | 2.06 |  | 1.30 |  |
|  | 200k> | 1.088 |  | 2.21 |  | 1.45 |  |

|  |  |  |  |  |  |  |  |  |  |
| --- | --- | --- | --- | --- | --- | --- | --- | --- | --- |
| **Table S6: Demographic characteristics and response rate proportion of population who joined before and after first wearable device (Fitbit) protocol was deployed (Nov 2020).** | | | | | | | | | |
|  | Groups (n) | Fitbit (before protocol launched) RR % | Fitbit (after protocol launched) RR % | P value |  |  |  |  |  |
| Ethnicity | Non- Hisp | 4.1 | 4.5 | 1 |  |  |  |  |  |
|  | Hisp | 1.26 | 1.29 |  |  |  |  |  |  |
| Race | White | 5.43 | 7.38 | 0.32 |  |  |  |  |  |
|  | African  American | 0.84 | 2.5 |  |  |  |  |  |  |
|  | Asian | 3.24 | 14.92 |  |  |  |  |  |  |
| Sex | Male | 2.66 | 5.58 | 1 |  |  |  |  |  |
|  | Female | 4.08 | 7.23 |  |  |  |  |  |  |
| Age Group | 18-44 | 3.64 | 7.19 | 0.92 |  |  |  |  |  |
|  | 45-64 | 3.54 | 6.25 |  |  |  |  |  |  |
|  | 65> | 3.31 | 6.01 |  |  |  |  |  |  |
| Income | <50k | 1.8 | 5.31 | 0.72 |  |  |  |  |  |
|  | 50k to 100k | 5.95 | 8.29 |  |  |  |  |  |  |
|  | 100k to 200k | 7.59 | 7.37 |  |  |  |  |  |  |
|  | 200k> | 6.86 | 8.09 |  |  |  |  |  |  |

| **Table S7: Demographic characteristics and response rate proportion of population who joined before and after EHR integration ('Sync4Science' protocol) was deployed.** | | | | | | | | | |
| --- | --- | --- | --- | --- | --- | --- | --- | --- | --- |
|  | Groups (n) | EHR (before sync4science) | EHR (after sync4science) | P value |  |  |  |  |  |
| Ethnicity | NonHispanic | 90.82 | 94.94 | 0.99 |  |  |  |  |  |
|  | Hispanic | 95.53 | 96.88 |  |  |  |  |  |  |
| Race | White | 89.28 | 94.08 | 1 |  |  |  |  |  |
|  | African  American | 96.88 | 97.67 |  |  |  |  |  |  |
|  | Asian | 89.11 | 91.53 |  |  |  |  |  |  |
| Sex | Male | 92.79 | 96.36 | 0.97 |  |  |  |  |  |
|  | Female | 91.02 | 94.66 |  |  |  |  |  |  |
| Age Group | 18-44 | 90.84 | 94.01 | 0.96 |  |  |  |  |  |
|  | 45-64 | 92.23 | 95.89 |  |  |  |  |  |  |
|  | 65> | 92.02 | 96.04 |  |  |  |  |  |  |
| Income | <50k | 94.66 | 96.62 | 0.99 |  |  |  |  |  |
|  | 50k to 100k | 89.16 | 93.44 |  |  |  |  |  |  |
|  | 100k to 200k | 87.02 | 92.62 |  |  |  |  |  |  |
|  | 200k> | 87.19 | 92.12 |  |  |  |  |  |  |
